# Supplementary material for: Predator avoidance promotes inter-bacterial symbiosis with myxobacteria in polymicrobial communities
Source: bioRxiv. 2026 Feb 13:2026.02.12.705600. Preprint. [Version 1] doi: 10.64898/2026.02.12.705600 (PMC12919014; doi:10.64898/2026.02.12.705600)
Supplement: Supplement 1 [file media-1.pdf]

Supplemental movies

Supplemental movie S1. Time-lapse of *A.* WIMSLP2/*M.* WIMSLP2 on TPM 1% agar pad after spot dried. Frame intervals 20 s over two hours with 60x objective. Arrows indicates lyses events of *Microvirga* (small rods) by *Archangium* (long rods). Some *Archangium* cells can be seen moving out of focus.

Supplemental movie S2. Time-lapse of WIMLSP2/*E. coli*-mCherry on TPM 1% agar pad after spot dried. Frame intervals 20 s over two hours with 60x objective. Arrows indicates lyses events of *E. coli*-mCherry (red) by *Archangium* (long rods).

Supplemental movie S3. Time-lapse of WIMLSP2/DK1622-GFP on TPM 1% agar pad after spot dried. Frame intervals 20 s over two hours with 60x objective. Arrows indicates lyses events of *Microvirga* (small rods) by DK1622-GFP (green).

Supplemental movie S4. Time-lapse of DK1622-GFP/ *E. coli*-mCherry on TPM 1% agar pad after spot dried. Frame intervals 20 s over two hours with 60x objective. Arrows indicates lyses events of *E. coli*-mCherry (red) by DK1622-GFP (green).
