# Supplementary material for: Predator avoidance promotes inter-bacterial symbiosis with myxobacteria in polymicrobial communities
Source: bioRxiv. 2026 Feb 13:2026.02.12.705600. Preprint. [Version 1] doi: 10.64898/2026.02.12.705600 (PMC12919014; doi:10.64898/2026.02.12.705600)
Supplement: Supplement 6 [file media-6.pdf]

Predator avoidance promotes inter-bacterial symbiosis with myxobacteria in polymicrobial communities

Shailaja Khanal*, Sheila Walsh**, Nawal Shehata, Andrew Ahearne, Daniel Belin, Britney Larson, Benjamin Tabor, Daniel Wall, and Cole Stevens

Supplemental Data

Supplemental Table S1: Assembly statistics for WIMLSP1

Scaffold ID (IMG/MER)	Sequence length (bp)	GC content	Gene count
Ga0669685_01	12,981,101	0.69	10,693
Ga0669685_02	2,513	0.64	6
Ga0669685_03	3,923,152	0.61	3,882
Ga0669685_04	2,538	0.68	2
Ga0669685_05	2,575	0.70	3
Ga0669685_06	2,776	0.69	2

Supplemental Table S2: Assembly statistics for WIMLSP2

Scaffold ID (IMG/MER)	Sequence length (bp)	GC content	Gene count
Ga0646599_01	12,928,281	0.69	10,576
Ga0646599_02	4,285,839	0.62	4,146
Ga0646599_03	5,931	0.56	13

Supplemental Table S3: Assembly statistics for FLWO

Scaffold ID (IMG/MER)	Sequence length (bp)	GC content	Gene count
Ga0654980_01	13,960,269	0.68	11,322
Ga0654980_02	2,321	0.65	2
Ga0654980_03	2,877	0.69	2
Ga0654980_04	1,081	0.66	3
Ga0654980_05	1,603	0.63	2
Ga0654980_06	1,183	0.64	1
Ga0654980_07	3,818,472	0.61	3,738
Ga0654980_08	1,347	0.67	1

Ga0654980_09	5,895	0.56	14

Supplemental Table S4: Assembly statistics for DLMAZ

Scaffold ID (IMG/MER)	Sequence length (bp)	GC content	Gene count
Ga0646598_01	44,387	0.58	50
Ga0646598_02	12,010,403	0.69	10,262
Ga0646598_03	19,741	0.71	23
Ga0646598_04	158,443	0.68	86
Ga0646598_05	4,194	0.68	7
Ga0646598_06	12,788	0.72	13
Ga0646598_07	133,616	0.59	113
Ga0646598_08	7,740	0.72	12
Ga0646598_09	6,295	0.70	9
Ga0646598_10	4,131,692	0.61	4,039

Supplemental Table S5: Swarm consortia details from assembled data

Swarm consortia	contigs	# of bases	# of RNA genes	# of protein coding sequences	Estimated # of genomes
WIMLSP1	6	16,914,655	182	14,387	2
WIMLSP2	3	17,220,051	181	14,534	2
FLWO	9	17,795,048	180	14,889	2
DLMAZ	10	16,529,299	156	14,442	2

Supplemental Table S6: dDDH data for swarm consortia myxobacteria. dDDH (d_4, in %) values provided by TYGS analysis of corresponding MAGs. dDDH values were not calculated for cells with "n.d."

	A. WIMLSP1	A. WIMLSP2	A.FLWO	C.DLMAZ	A.gephyra	A.lansingense	C.fuscus
**A.WIMLSP1**	100	92.7	34.8	25.3	45.9	36.2	24.9
**A.WIMLSP2**	92.7	100	34.8	25.1	45.7	36.2	24.9
**A.FLWO**	34.8	34.8	100	25.2	35.4	44.9	24.9
**C.DLMAZ**	25.3	25.1	25.2	100	25.2	25.9	52.3
**A.gephyra**	45.9	45.7	35.4	25.2	100	n.d.	n.d.
**A.lansingense**	36.2	36.1	44.9	25.9	n.d.	100	n.d.
**C.fuscus**	24.9	24.9	24.9	52.3	n.d.	n.d.	100

Supplemental Table S7: dDDH data for swarm consortia *Microvirga*. dDDH (d_4, in %) values provided by TYGS analysis of corresponding MAGs. dDDH values were not calculated for cells with "n.d."

	M. WIMLSP1	M. WIMLSP2	M.FLWO	M.DLMAZ	M.guangxiensis	M.solisilvae	M.vignae
**M.WIMLSP1**	100	25.9	38	25.9	30.3	28.5	27.8
**M.WIMLSP2**	25.9	100	25.6	38.5	24.9	25.3	24.8
**M.FLWO**	38	25.6	100	25.8	30.2	28.5	27.7
**M.DLMAZ**	25.9	38.5	25.8	100	25.2	25.5	24.9
**M.guangxiensis**	30.3	24.9	30.2	25.2	100	n.d.	n.d.
**M.solisilvae**	28.5	25.3	28.5	25.5	n.d.	100	n.d.
**M.vignae**	27.8	24.8	27.7	24.9	n.d.	n.d.	100

Supplemental Table S8: Genes that are phylogenetically associated with Myxococcota that are present in *Microvirga* MAGs.

MAG	AA sequence	annotation	Top BLASTP hit (species	2nd BLASTP hit (species)
**M.WIMLSP1**	MLKLAFAAAVGTTLLFASGAQALETQNGLAFNGLA FNGLAFNGLAFNGLAFNGAAADGVAGELRSAPALQ ATTVILKDGERVSLK	hypothetical protein	Microvirga sp. ACRRW	Pyxidicoccus sp. 3LG
	MLMNQDEYDKHLKGFMITGCTVRSKDVFYLVAITD SPNRARPESDLTTRVIPYFFEKIEKRWGHINYHGYS RTLAGASLYPESKFVGVDRGGQVMVVGGGKMEIE DIAGGRTGPIRGSVNRVRTINGFIHVCSNNRGLARR DGTDRWTSLCADLPVKPNPNGFGEVYGFNDFDAF DNGEFYCVGGQSDVWRFDGANWTPIDVPGDHNG ASNFLTKAGSKIARVPLHSVCCAGDGYLYIGGPDG GVWKGRNEQWKLIHDDRLSLPFKDIVWFQDRVYC TSRYGLWEIVNDEVRPCDVPEEISICSGNLAVADGI MLLAGECGAAYHDGREWKLIFNTSSFT	WD40/YVTN/BNR-like beta propeller repeat protein	Microvirga sp. ACRRW	Archangium sp.
	MIFRHLRGGLRAVLFTLLGLLGYPSLVSAAGPDMYF DAPADKQMALAVASGDIETMTALLSSKAVDPQAIG RKATSWIEIAIIADQKKAFDTLMKWNALGPPKGKIA GQAMYSATVKGSIRWLERLAAAGASLDNYGGGEL LIVTALDTRNEAVLDFYIRNGADLDMPAMAGGSVAL SAAMTRRFDMALRFLDLGASPWVMDSLGSTLGSIA ERAARVPAWDHSSRMNQHRLELLQRLHAIGFPDP APTADEGHALRQKKQWPPKAAIKQ	ANKYR superfamily	Microvirga sp. ACRRW	Archangium sp.
	MFKIPEPMLTYWASKAYKKASIADLAEVERLFGTAL PASYVEFVTTIGFVVFDDVPGFKIHEYFDYKVGSPE GTEIAQGNIAFLKEPAHIIKAHKILTNRQALEEEEEED EDFPKFPKNYLPIANDAGQGQILMEFGEHPGRIWY WQENDWAWGLEDNTWLGFVAENFEDFINGLKP	SM1/KNR4 family protein	Archangium violaceum	Candidatus Methylumidphilus sp.
**M. WIMLSP2**	MSRTMKYTILVSFVGLLSAGAQAAEIANGSDLNGA NLNGANLNGSDLNGSDLNGANLNGASSGRVFLGA QVSALIAPDGTLVTLD	pentapeptide repeat protein	Microvirga sp.	Archangium violaceum
	MMTTIDDIRRRDGFLTIEETLSLCDRNTIYDPYSTLIS RHARIGSGNILYPCTTIRCSQDSSCEVGDRNIFHSL TMIDACGGAISIGSGNTFGDGGFTAKADRPGAKITI GDRGRYASGASVYGVSHLGTGSQILGQISVIDCVL ADGEDFTHADPDERAAVLKGHGAARKLRIGVGEVI FGKSSFDQAGIQRQTDFHPKS	IpxD-like protein	Pyxidicoccus sp. 3LG	Stappiacea bacterium
	MSYPFYLLYGAYLASGLGDWILRIAVPLLIYQVTDSA LAMAGAYAVNYLPYLIVTPFGGVLADRVDRRRMLL VGDFFAAGLVVAIILANASGAAALLLYPLLFVLASTA AVYHPGFQSFIPSVVPPDKLARANSLFAAADNGLSL LGPAAAGGIVALLGPVQALYADALSFALSGLLILCIP SAMSAKAQVERKLQGILHDLREGFVYVWHNRILRA GAFLFFFVNFSYDIFYANFIFLLVGIFGLTAVDAGTVI SMTGVGALVGSLVAPKLMSRVSSGRLIVACTATAG GLILLLLFVDGALAVGMLWGGVCATQAVIRVAYFTL RQKIVPSNLLGRSVAVTRMISYAAVPLAALSGGWIV QQTGEIRMIVIISGSVMLLSALIAWFTSLGRTPQPAL QSSLAT	MFS transporter	Microvirga sp.	Polyangium aurulentum
	MILRQLRGGLRALLFMLLGLFGYPASASAAGPDMY FDSPADKQMALAVASGDIETMTALLSSKAVDPLAIG RKVTSWIEIAVIADQRAAFDALVKWGALGPAKGKIA GQAMYSATIKGSIRWLERLTAAGASLDNHGGGDLL IVQALDTRNEAVLDFYIRNGADLNMPAMAGGSVAL SAAMTRRFDMVLKFLDLGASPWVMDSLGSTLGSIA	ANKYR superfamily	Microvirga sp. ACRRW	Archangium sp.

	ERAARVPAWDHSSRMNQHRLELLRRLHAIGFPDPAPTANEGHALRQKKQWPPKAAIKQ			
**M. FLWO**	MRHRISIGLACILCLMGAGCLDEREKAARTLVKTRACPDCDLTEIKLEAAQLQGAQLAGARLEKADMRKADLREADFSGAILFDTDLRGADLRGAAFREASMTGAQMQGANLEGVDLSGTTLNAIDLSGVNFQGANLRGAKLSEARLNGINGPYRRDPPAHFYAVPAGGADLRGADLSGADLSGAYLSKADLRDAKLAGANLRDAHLDQADLRGADINGADLKGAILDHATWIDGSICAEKSIGRCRRP	pentapeptide repeat protein	Corallococcus sp.	Corallococcus silvisoli
	MKASKWQLKQRWHEEAGALVLKRVQDILLADDTRKLPLGIPDILSGLPFRDEVASGRDFRGIELEGGLTSLDLSGCDFSYAKLTLNFIRCDLSEANFEEATLGGIIFDKATRANFRRAKMRHCSLVGLNAQDCCFDEAILSNASFEKACLQGSKFRNANCKGASFVSANLLGCDFQGANLNECPFQGVILDRSTNLRGASLVGLFYHEHRSIDGKLVLPKTDWRLATHDETTRTEA	pentapeptide repeat protein	Hyalangium gracile	Archangium sp.
	MLKTAFAATVGTTLLFGSGAQALETQNGLAFNGLAFNGLAFNGLAFNGLAFNGAATDGVAEELRSAPALQATTVILKDGEHVSLK	hypothetical protein	Microvirga sp. ACRRW	Pyxidicoccus sp. 3LG
**M. DLMAZ**	MACAIGLYHRVKARIPTIAVICTDANSCYRLAFARHGVAEAHVQSKAHTH	transposase	Corallococcus sp. AB045	Alphaproteobacteria bacterium
	MTCHHCGSTAFRKNGHCAGVQRYVCHACHRSFSANGERFSKAVKAQALDM	transposase	Corallococcus sp. AB045	Accumulibacter sp.
	MGVLERLVLTDSQWARIAPLIIGRPDQKGSTGRDNRMFVEGVLWIVRTGS	transposase	Microvirga sesbaniae	Corallococcus sp. AB045
	MACHHCGSSAFCKNGHTRGVQRYRCHACHRSFSANGERFSKTVKAQALDMYLNNVGLRKIARFTGASPPAVLKWIKKAATALAAQLEQAKAQVHDELPDVIEMDEIYTFVQKNSSAPSYGLLILDGRAVLLRTSSATGA	transposase	Corallococcus sp. AB045	Accumulibacter sp.
	MSSAIGLYRRVKQAVPAVALICTDANSCYRLAFERYRVPEAHVQSKAHTH	transposase	Corallococcus sp. AB045	Mesorhizobium sp. 8
	MLLDQTAYDAYFKGFILIDCVIRSKDIFYFVLVSDLKRTRSESDRKTRIVAHFLKSSDKPWRRADYEGFAKVFAGASQLPASKFVGVDRGAQVMLIGSGSLENEDIPAGKQGPIRGAIRKIKTINGYAHVCSGYRGFARRDGPNLWTSLVKNLNFMPDPDKDSGIYGFADFDAFNDRDFYCVGGHSDAWHFDGETWTQLDFPGDPSQIPESLIDPSTPGVPLEAVCCAGNGYVYIGGPGGTVWKGRKNSWTLIHRDSMSLPLRDMVWFKDRVYCTSDYGLWEIVDDQLRPCDIPDEIRVCSGHLSVCDGVMLLAGIYGAAYHDGNRWHLIFDTGQF	WD40/YVTN/BNR-like beta propeller repeat protein	Microvirga sp. ACRRW	Archangium sp.

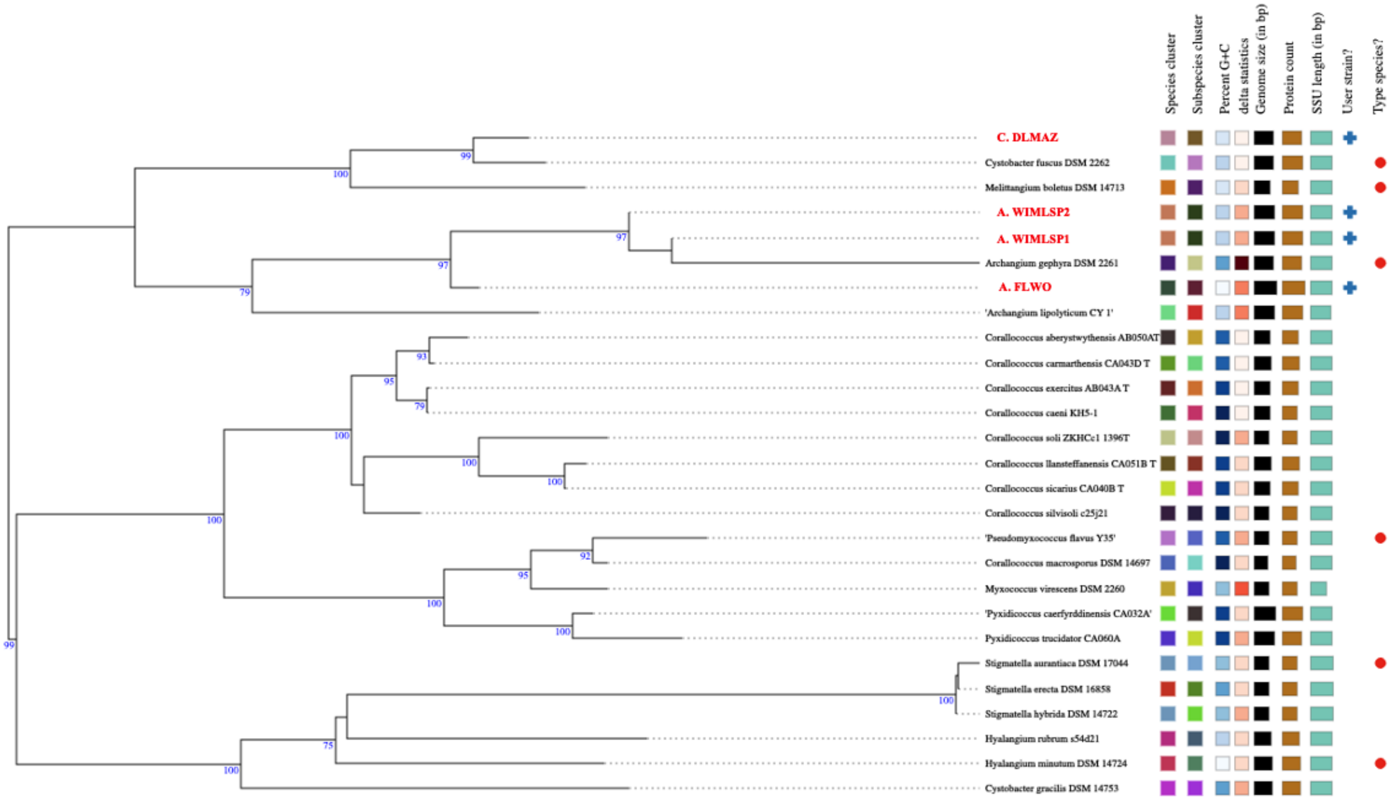

Supplemental Figure S1: Genome BLAST Distance Phylogeny (GBDP) tree generated from myxobacterial 16S rDNA gene sequences at the Type Strain Genome Server (TYGS).

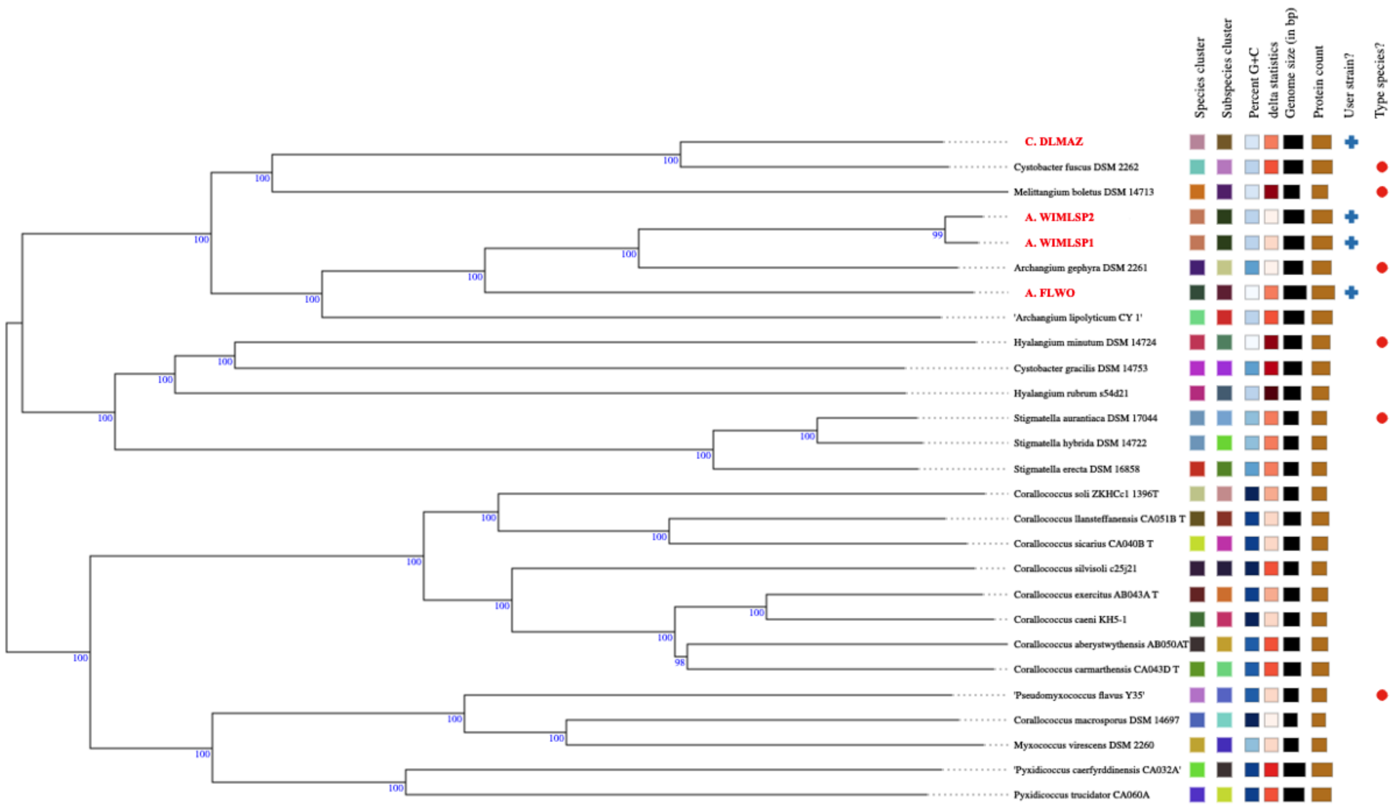

Supplemental Figure S2: Genome BLAST Distance Phylogeny (GBDP) tree generated from myxobacterial MAG sequences at the Type Strain Genome Server (TYGS).

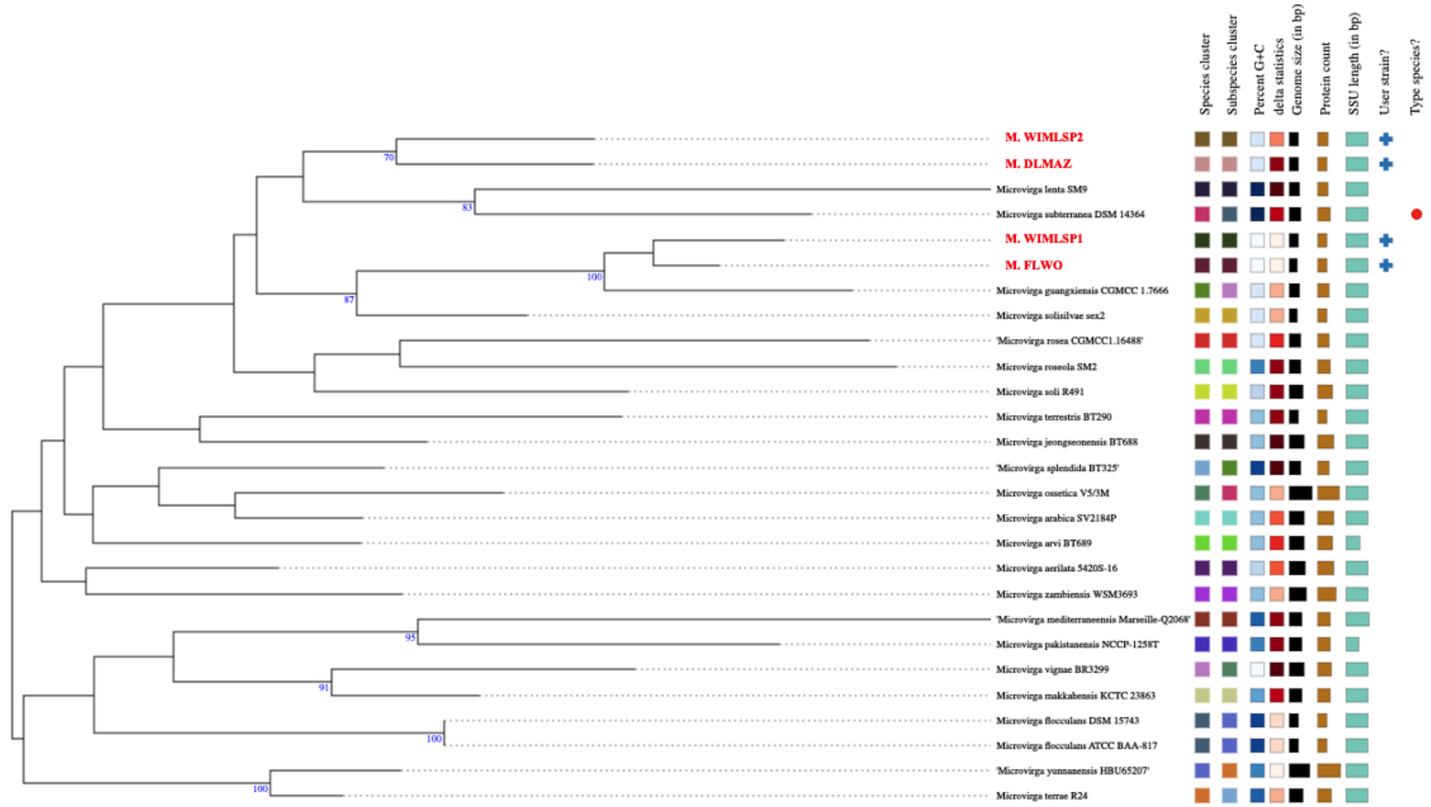

Supplemental Figure S3: Genome BLAST Distance Phylogeny (GBDP) tree generated from *Microvirga* 16S rDNA gene sequences at the Type Strain Genome Server (TYGS).

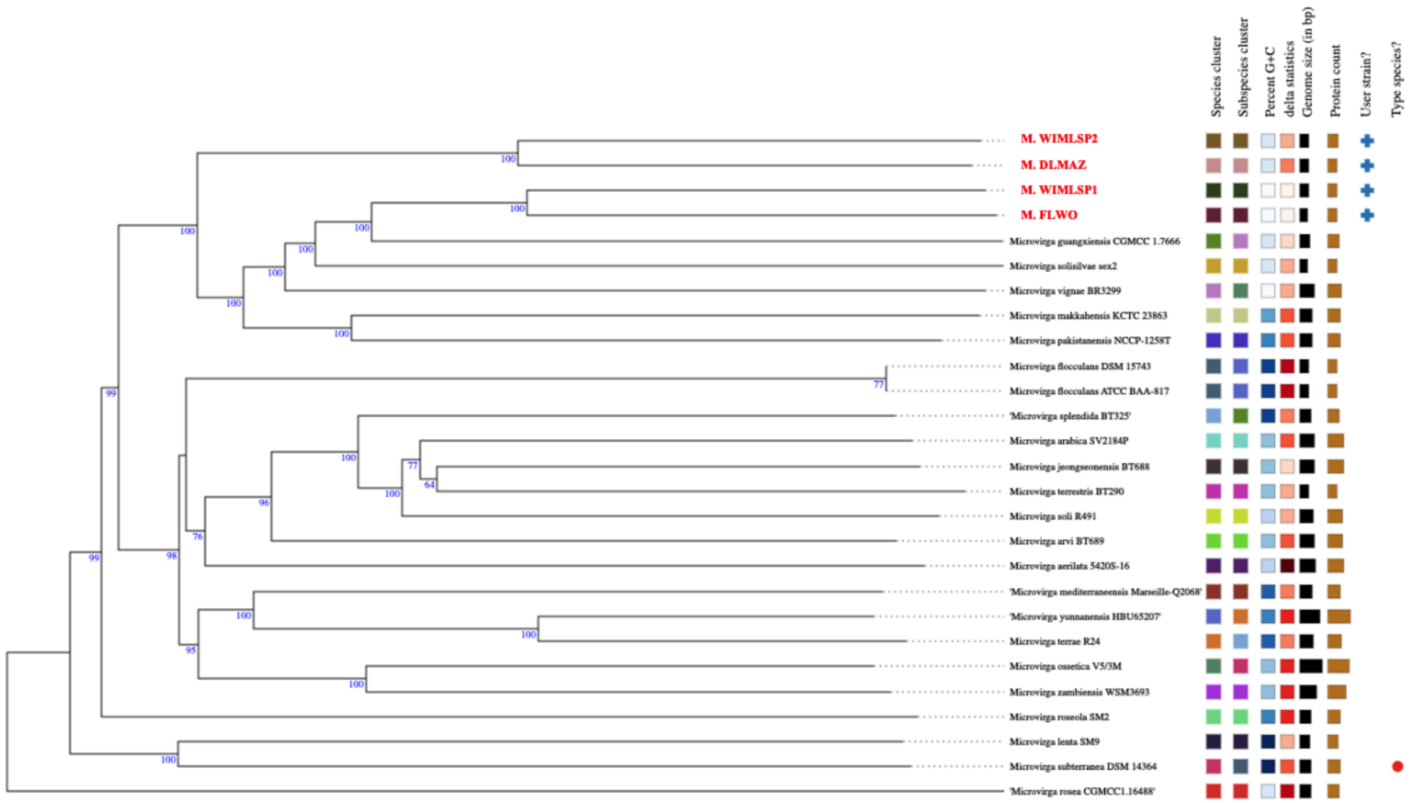

Supplemental Figure S4: Genome BLAST Distance Phylogeny (GBDP) tree generated from *Microvirga* MAG sequences at the Type Strain Genome Server (TYGS).

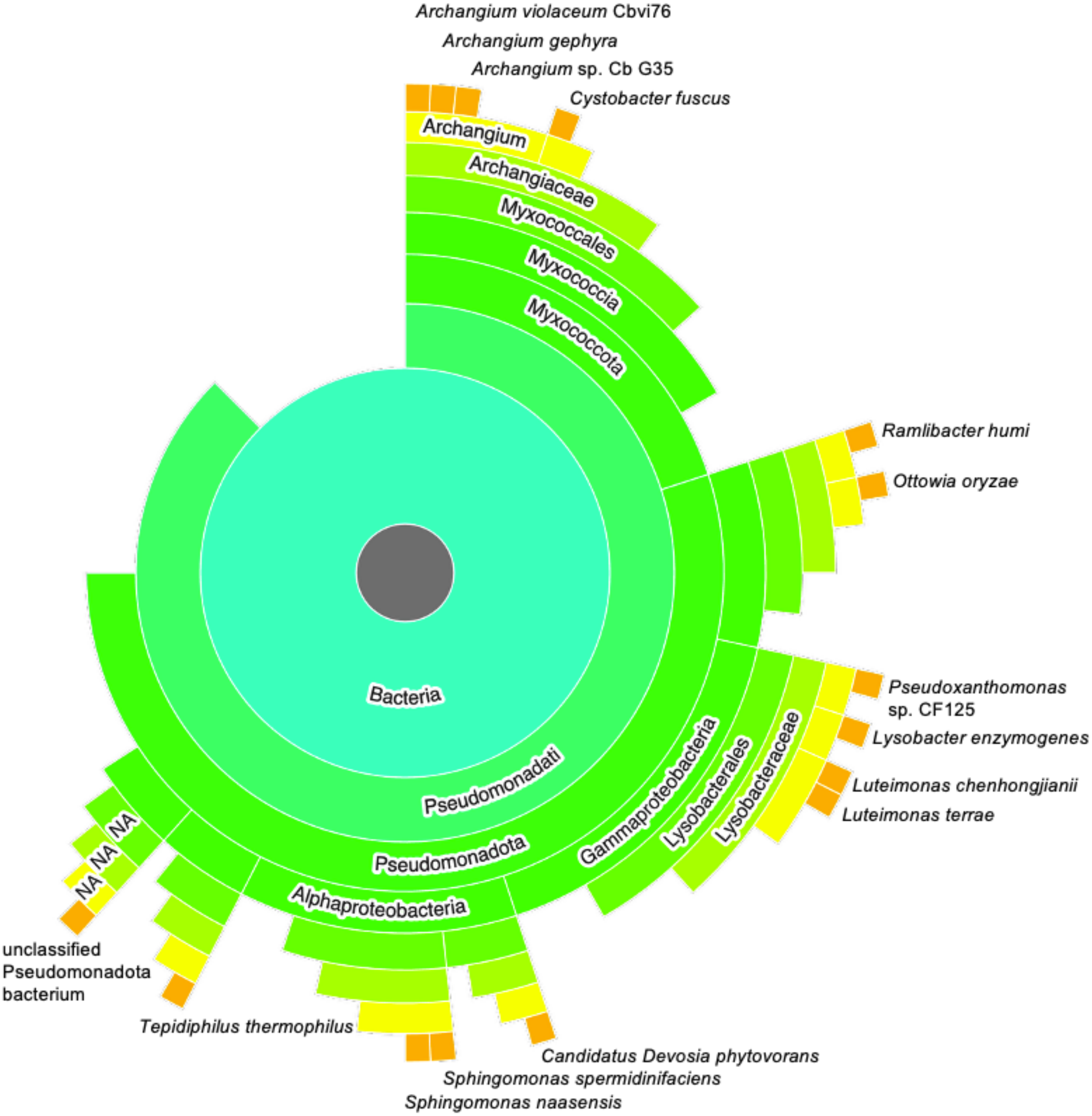

Supplemental Figure S5: Phylogenetic distribution of ANKYR proteins identified with EFI-EST analysis.

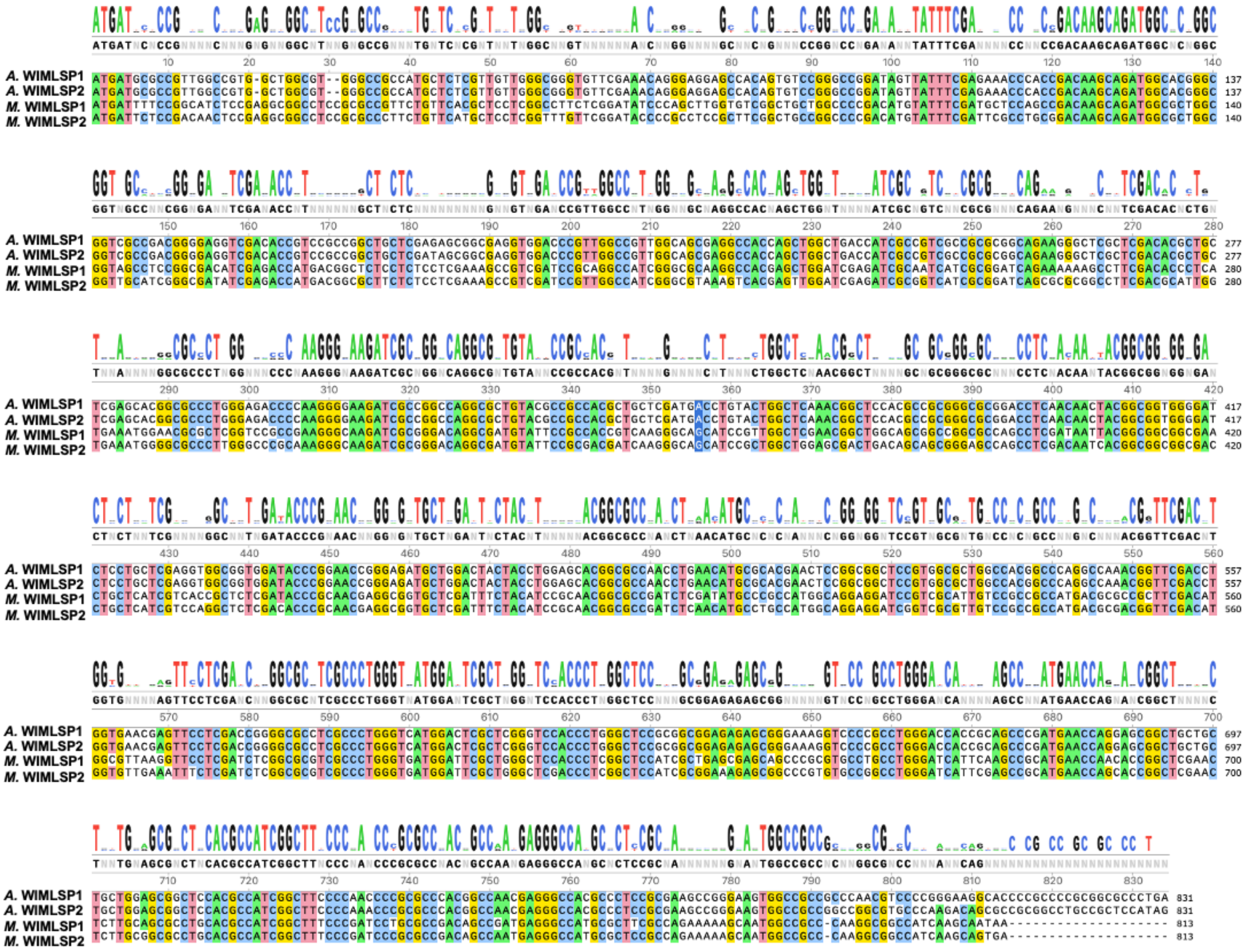

Supplemental Figure S6: Alignment of genes encoding ANKYR proteins from WIMLSP1 and WIMLSP2 generated with MEGA X using Clustal.

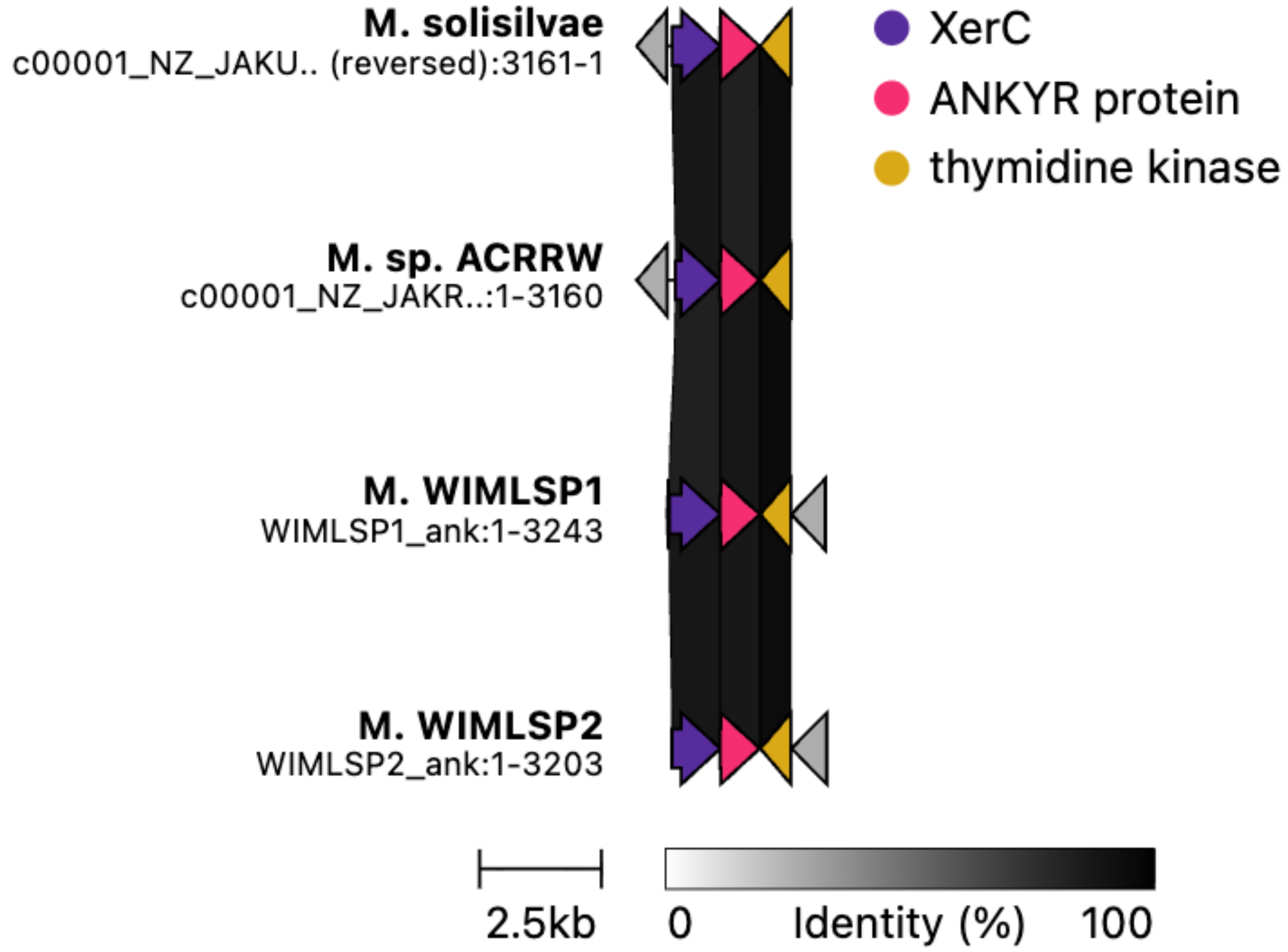

Supplemental Figure S7: Conserved spatial organization of XerC and ANKYR proteins in genomes of *M.* WIMLSP1, *M.* WIMLSP2, *M. solisilvae*, and *M.* sp. ACRRW. Figure rendered with clinker.

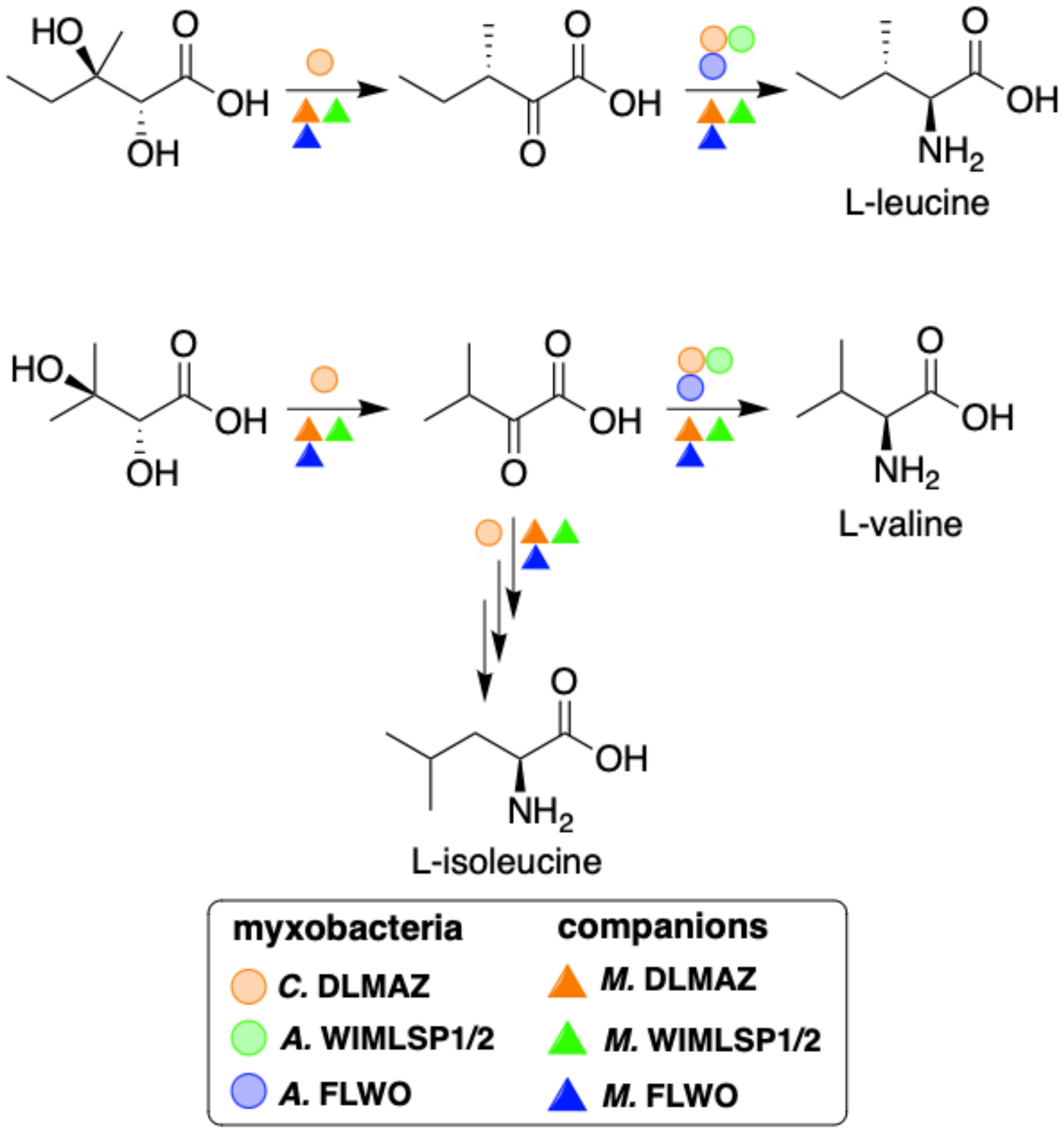

Supplemental Figure S8: BCAA biosynthetic pathways from swarm consortia depicting BCAA auxotrophy in *Archangium*.
